# Stability analysis and selection of sugar beet (*Beta vulgaris* L.) genotypes using AMMI, BLUP, GGE biplot and MTSI

**DOI:** 10.1038/s41598-023-37217-7

**Published:** 2023-06-20

**Authors:** Dariush Taleghani, Abazar Rajabi, Ali Saremirad, Parviz Fasahat

**Affiliations:** Sugar Beet Seed Institute (SBSI), Agricultural Research, Education and Extension Organization (AREEO), Karaj, Iran

**Keywords:** Plant breeding, Plant development, Plant genetics

## Abstract

The methods utilized to analyze genotype by environment interaction (GEI) and assess the stability and adaptability of genotypes are constantly changing and developing. In this regard, often instead of depending on a single analysis, it is better to use a combination of several methods to measure the nature of the GEI from various dimensions. In this study, the GEI was investigated using different methods. For this purpose, 18 sugar beet genotypes were evaluated in randomized complete block design in five research stations over 2 years. The additive effects analysis of the additive main effects and multiplicative interaction (AMMI) model showed that the effects of genotype, environment and GEI were significant for root yield (RY), white sugar yield (WSY), sugar content (SC), and extraction coefficient of sugar (ECS). The multiplicative effect's analysis of AMMI into interaction principal components (IPCs) showed that the number of significant components varies from one to four in the studied traits. According to the biplot of the mean yield against the weighted average of absolute scores (WAAS) of the IPCs, G2 and G16 for RY, G16 and G2 for WSY, G6, G4, and G1 for SC and G8, G10 and G15 for ECS were identified as stable genotypes with optimum performance. The likelihood ratio test showed that the effects of genotype and GEI was significant for all studied traits. In terms of RY and WSY, G3 and G4 had high mean values of the best linear unbiased predictions (BLUP), so they were identified as suitable genotypes. However, in terms of SC and ECS, G15 obtained high mean values of the BLUP. The GGE biplot method classified environments into four (RY and ECS) and three (WSY and SC) mega-environments (MEs). Based on the multi-trait stability index (MTSI), G15, G10, G6, and G1 were the most ideal genotypes.

## Introduction

Climatic conditions along with soil characteristics are the main environmental factors that affect the plants’ growth and also the agricultural productivity^1^. Therefore, it is necessary to consider the effect of environment as many environmental factors overshadow the genetic potential of the plant and cause low performance in commercial fields^2^. The yield gap between measured yield in cultivar introduction trials and actual yield in farmers' fields has been estimated to be more than 30% in some countries^3^. The main reason is the lack of yield stability as a result of GEI. It is important to note that with increase in the genetic potential of the yield, the plant will naturally have a greater demand for the agricultural resources and as a result, it is subjected to the risk of biotic and abiotic stresses. Therefore, high yield potential should be accompanied by increased tolerance against unfavorable environmental factors; as a result, the yield stability does not decrease with an increase of yield potential.

GEI refers to the different response of genotypes in a wide range of environments. It is known by researchers who are active in the field of crop breeding as it affects breeding progress and makes it difficult to evaluate and select superior genotypes. Therefore, it is of importance to breeders in terms of development and release of new high-yielding cultivars. Meanwhile, quantitative traits such as yield, which are economically and agronomically important, can be significantly affected by GEI. This force breeders to be more careful in evaluating and releasing genotypes and choose the best genotypes in terms of yield and adaptability in target environment. On the other hand, GEI may also provide opportunities for breeders to select genotypes that interact positively with a particular location (specific adaptation) or perform well in most environments (general adaptation). This can be achieved by cultivating genotypes in different environments, recording their response, and finally selecting a superior and stable genotype^4^.

High yield potential in sugar beet is vital for efficient productivity in arable land. Sugar beet is one of the most important sugar crops supplying about 30% (nearly 42 million tons) of the world's sugar needs^5^ and is known as the second sugar crop after sugarcane^6,7^. Sugar beet genotypes adapt to different environments in a different way, so their evaluation can be different according to the breeding goals. These assessments require a series of multi-environment trials (METs) in the advanced selection phase to be valid^8^. Each genotype should be evaluated for several years in different locations in order to identify high yielding and stable genotypes and recommend them for cultivation in target areas. Therefore, METs are carried out to evaluate genotypes for more reliable results. By cultivating genotypes in different environments, different genotypic responses are recorded, and a superior and stable genotype(s) can be identified.

Several methods are introduced to analyze the information obtained from METs and are generally divided into two main groups of univariate and multivariate methods. Among the multivariate methods, AMMI model is of great importance. AMMI method is actually a combination of analysis of variance and principal components (PCs) analysis. The first part of AMMI, i.e., the additive part, uses analysis of variance^9^ and the second part, which includes the multiplicative part, uses the PCs analysis method in order to analyze the GEI based on PCs^10^. In fact, the reason for the widespread use of this model is the high resolution of main effects and GEI, as well as the justification of a large part of the sum of squares of the interaction^11^. Recently, a new index called the WAAS^12^ has been added to the indices based on the AMMI model. In this approach, all the IPCs (in addition to first and second components) are considered and used to draw the biplot of the average performance against the first PC of the GEI (AMMI1). As a result, the variance of the GEI is fully included in the selection of the superior genotype^12^.

The AMMI model is often used together with the GGE biplot graphical model to identify MEs as well as winning genotypes in each ME. This graphical model has been proposed based on PC analysis^10,13–15^. This method helps breeder to simply evaluate the genotype stability and the combination of stability with the yield of genotypes and different environments through the graphical representation of GEI. The unique feature of GGE biplot is that based on the plots, it can be decided which genotype has the highest potential in which environment or subgroup^16^. In addition to the above-mentioned methods, BLUP has also been used to analyze METs^17^. This method estimates the mean yield of genotypes in mixed models with high efficiency. In order to use the advantages of both AMMI and BLUP, an index called weighted average absolute scores of BLUPs (WAASB) has been introduced, which is actually the integration of AMMI and BLUP^12^. Since, in identifying and introducing new cultivars, breeders consider both stability and yield characteristics at the same time, in addition to reduce the GEI, genotypes with high yield potential are selected; therefore, considering the WAASB index and yield (Y), the WAASBY index was introduced, in which both stability and yield characteristics are taken into account at the same time^12^.

Recently, Olivoto, et al.^12^ introduced the theoretical basis of the MTSI to select high yield and stable genotypes in METs based on multiple traits considering both fixed and random effects models. The MTSI is calculated based on the distance from the ideal genotype estimated through factor analysis. This index provides the possibility of selecting stable genotypes with a positive selection differential for traits that are intended to increase and a negative selection differential for traits that are intended to decrease. On the other hand, this stability index can be useful for breeders whose goal is to simultaneously select for average performance and stability by considering several traits; because it provides a unique selection process that is easy to interpret and takes into account the correlation structure among traits^12^.

In this study, a set of new sugar beet hybrids were cultivated with the aim of evaluating the effect of GEI on the potential for quantitative production of sugar and also determining their adaptability in a wide range of environments in order to identify superior hybrids.

## Materials and methods

### Plant materials

In the present study, nine selected pollinator lines of sugar beet from different sugar content groups, were crossed with two single crosses in 2019. A total of 18 sugar beet genotypes (Table 1) were evaluated in field experiments. Genotypes were obtained from the Seed Bank of the Sugar Beet Seed Institute (SBSI), Karaj, Alborz, Iran.

**Table 1:** Characteristics of the studied sugar beet genotypes.

Row	Genotype code	Origin		Row	Genotype code	Origin	
		♀	♂			♀	♂
1	G1	F-21121	SB27	10	G10	F-21122	SB33
2	G2	F-21122	SB27	11	G11	F-21121	SB51
3	G3	F-21121	920,760	12	G12	F-21122	SB51
4	G4	F-21122	920,760	13	G13	F-21121	HSF-14-P.35
5	G5	F-21121	S1-24	14	G14	F-21122	HSF-14-P.35
6	G6	F-21122	S1-24	15	G15	F-21121	950,123
7	G7	F-21121	920,128	16	G16	F-21122	950,123
8	G8	F-21122	920,128	17	G17	F-21121	33,866-94
9	G9	F-21121	SB33	18	G18	F-21122	33,866-94

### Location and field experimental design

Field experiments were conducted over 2 years (2020–2021) in five research stations in a randomized complete block design with four replications. The characteristics of the environments and physicochemical properties of the soil are presented in Tables 2 and 3, respectively. The experimental plots used in the study had six rows, each of which was 10 m long with a 0.5-m interrow spacing.

**Table 2:** Geographical characteristics of the experimental environments.

Row	Environment code	Year	Location of the research station	Altitude (m)	Latitude	Longitude
1	E1	2020	Karaj, Alborz, Iran	1312	35° 55ʹ N	50° 54ʹ E
2	E2	2021				
3	E3	2020	Mashhad, Khorasan Razavi, Iran	1316	36° 30ʹ N	59° 37ʹ E
4	E4	2021				
5	E5	2020	Shiraz, Fars, Iran	1484	29° 32ʹ N	52° 36ʹ E
6	E6	2021				
7	E7	2020	Miandoab, West Azerbaijan, Iran	1296	36° 58ʹ N	46° 05ʹ E
8	E8	2021				
9	E9	2020	Hamedan, Hamedan, Iran	1818	34° 47ʹ N	48° 30ʹ E
10	E10	2021

**Table 3:** Physicochemical properties of soil at the experimental environments.

Environment code	pH	EC (ds m^−1^)	O.C. (%)	P (ppm)	K (ppm)	Clay (%)	Silt (%)	Sand 9%)	Texture
E1	7.21	0.53	0.74	8.24	596	37.30	41.40	21.30	Clay-loam
E2	7.30	0.77	1.01	1.97	398	37.30	41.40	21.30	Clay-loam
E3	7.90	1.55	0.70	12.00	261	21.00	57.00	20.00	Silty-clay-loam
E4	7.65	1.67	0.75	12.10	296	16.00	36.00	48.00	Silty-loam
E5	8.00	0.93	0.74	1.02	490	36.00	43.20	20.80	Clay-loam
E6	7.80	2.01	0.76	4.40	264	36.00	43.20	20.80	Clay-loam
E7	7.30	0.83	49.00	13.00	402	65.00	58.00	25.00	Silty-loam
E8	7.10	0.78	56.00	17.00	521	61.10	55.40	23.70	Silty-clay-loam
E9	7.93	6.14	0.45	47.60	499	15.50	27.50	53.00	Silty-loam
E10	7.93	6.14	0.45	47.60	499	15.50	27.50	53.00	Silty-loam

### Measurement of traits

At harvest, roots of the experimental plots were weighed and RY was estimated. After washing the roots, pulp samples were randomly taken and examined for qualitative characteristics at the Quality Control laboratory of Sugar Beet Seed Institute (SBSI). These quality characteristics included SC, alpha amino N, sodium (Na^+^), and potassium (K^+^) elements^18^. Based on the values obtained for these properties, WSY, and ECS were estimated by Eqs. (1) and (2), respectively^19,20^. However, all international, national and institutional guidelines have been taken into account in various stages of experiments.

1 $$WSY=WSC\times RY$$

2 $$ECS=\left(\frac{WSC}{SC}\right)\times 100$$

where WSY is white sugar yield (t ha^−1^), WSC is white sugar content (%), RY is root yield (t ha^−1^), ESC is extraction coefficient of sugar (%) and SC is sugar content (%).

### Statistical analysis

The homogeneity of the variances of experimental errors was checked with Bartlett's test^21^. After the homogeneity of error variances was confirmed, a variance analysis based on the AMMI model was performed on each trait's data. Equation (3) was used to perform stability analysis by the AMMI method^9^.

3 $${Y}_{ge}=\mu +{\alpha }_{g}+{\beta }_{e}+\sum_{n} {\lambda }_{n}{\gamma }_{gn}{\delta }_{en}+{\rho }_{ge}$$

where ${Y}_{ge}$ is the yield of genotype *g* in environment *e*; $\mu$ is the grand mean; ${\alpha }_{g}$ is the genotype deviation from the grand mean; ${\beta }_{e}$ is the environment deviation; ${\lambda }_{n}$ is the singular value for *IPC*_*n*_ and correspondingly ${\lambda }_{n}^{2}$ is its eigenvalue; ${\gamma }_{gn}$ is the eigenvector value for genotype *g* and component *n*; ${\delta }_{en}$ is the eigenvector value for environment *e* and component *n*, with both eigenvectors scaled as unit vectors; and ${\rho }_{ge}$ is the residual. By performing AMMI analysis of variance using R software (metan package), the IPCs were obtained for each genotype and environment. WAAS were estimated from the IPCs of AMMI analysis of variance. The IPC1 in the traditional AMMI1 biplot was replaced by WAAS.

4 $${\mathrm{WAAS}}_{\mathrm{i}}=\frac{\sum_{\mathrm{k}=1}^{\mathrm{P}} \left|{\mathrm{IPCA}}_{\mathrm{ik}}\times {\mathrm{EP}}_{\mathrm{k}}\right|}{\sum_{\mathrm{k}=1}^{\mathrm{P}} {\mathrm{EP}}_{\mathrm{k}}}$$

where ${\mathrm{WAAS}}_{\mathrm{i}}$ is the weighted average of absolute scores of the ith genotype or environment; ${\mathrm{IPCA}}_{\mathrm{ik}}$ is the absolute score of the ith genotype or environment in the kth IPC; and ${\mathrm{EP}}_{\mathrm{k}}$ is the magnitude of the variance explained by the kth IPC.

To evaluate the genotypes stability across the environments, a linear mixed model was used. The variance components were estimated by restricted maximum likelihood. The estimations were performed assuming genotype and GEI as random effects. The significance of random effects was tested by the likelihood ratio test. The BLUP of ith genotype was predicted as the sum of the general mean overall environments and genotypic effect. WASSB were estimated based on single value decomposition of the GEI effects from the matrix of the BLUP. The equation of WAASB (5) is similar to WAAS. The WAASBY was calculated allowing weighting between its mean performance and stability based on Eq. (6):

5 $${\mathrm{WAAS}B}_{\mathrm{i}}=\frac{\sum_{\mathrm{k}=1}^{\mathrm{P}} \left|{\mathrm{IPCA}}_{\mathrm{ik}}\times {\mathrm{EP}}_{\mathrm{k}}\right|}{\sum_{\mathrm{k}=1}^{\mathrm{P}} {\mathrm{EP}}_{\mathrm{k}}}$$

6 $${\mathrm{WAAS}BY}_{\mathrm{i}}=\frac{\left({rG}_{g}\times {\theta }_{Y}\right)+({rW}_{g}\times {\theta }_{S})}{{\theta }_{Y}+{\theta }_{S}}$$

where (Eq. 5) ${\mathrm{WAASB}}_{\mathrm{i}}$ is the weighted average of absolute scores of the ith genotype or environment; ${\mathrm{IPCA}}_{\mathrm{ik}}$ is the absolute score of the ith genotype or environment in the kth IPC; and ${\mathrm{EP}}_{\mathrm{k}}$ is the magnitude of the variance explained by the kth IPC. In Eq. (6), ${\mathrm{WAAS}BY}_{\mathrm{i}}$ is the superiority index with different weights between yield and stability for the gth genotype; ${\theta }_{Y}$ and ${\theta }_{S}$ are the weights for yield and stability, respectively; ${rG}_{g}$ and ${rW}_{g}$ are the rescaled values of the gth genotype for yield and WAASB, respectively.

Graphic analysis of GGE biplot was done based on single value decomposition according to Eq. (7):

7 $${Y}_{\mathrm{ij}}-\mu -{\beta }_{\mathrm{j}}={\lambda }_{1}{\xi }_{\mathrm{i}1}{\eta }_{\mathrm{j}1}+{\lambda }_{2}{\xi }_{\mathrm{i}2}{\eta }_{\mathrm{j}2}+{\varepsilon }_{\mathrm{ij}}$$

where, ${Y}_{\mathrm{ij}}$ is the mean of ith genotype in jth environment, $\mu$ is the mean of all genotypes, ${\beta }_{\mathrm{j}}$ is the main effect of jth environment, ${\lambda }_{1}$ and ${\lambda }_{2}$ are the special quantities for the first and second components, respectively, ${\xi }_{\mathrm{i}1}$ and ${\xi }_{\mathrm{i}2}$ are the special vectors of genotypes, and ${\eta }_{\mathrm{j}1}$ and ${\eta }_{\mathrm{j}2}$ are the environmental vectors of first and second components, respectively, and ${\varepsilon }_{\mathrm{ij}}$ is the remaining quantity for the ith genotype in jth environment.

MTSI was computed to calculate the mean performance and simultaneous stability of RY, WSY, SC and ECS based on Eq. (8)^12^.

8 $${MTSI}_{i}={\left[\sum_{j=1}^{f} {\left({\gamma }_{ij}-{\gamma }_{j}\right)}^{2}\right]}^{0.5}$$

where ${MTSI}_{i}$ is the multi-trait stability index of the genotype *i*, ${\gamma }_{ij}$ is the score of the genotype *i* in the factor *j*, and ${\gamma }_{j}$ is the score of the ideal genotype in the factor *j*. Scores were calculated based on factor analysis for genotypes and traits.

## Results and discussion

### Additive main effects and multiplicative interaction (AMMI)

Considering that the error variance of different trials was homogeneous, the analysis of variance of the AMMI's model additive effects was performed for RY, WSY, SC and ECS (Table 4). The significant effect of the environment at the 1% probability level for all above-mentioned traits indicated the existence of a difference among the experimental environments. The genotype effect was also significant for all studied traits at the 1% probability level; therefore, the studied genotypes showed different RY, WSY, SC and ECS. GEI was also significant for all traits at the 1% probability level. It caused variation in genotypes ranking in different environments for all studied traits. Such a result has been reported in almost all similar studies^22–25^.

**Table 4:** Additive effects analysis of variance of AMMI model for the studied traits of sugar beet genotypes.

Source of variation	df	Mean of squares			
		Root yield (RY)	White sugar yield (WSY)	Sugar content (SC)	Extraction coefficient of sugar (ECS)
Environment	9	13,998.20**	338.67**	275.95**	663.73**
Error 1	30	247.00	6.90	3.85	44.71
Genotype	17	1343.90**	42.61**	7.88**	95.03**
Environment: genotype	153	249.70**	6.31**	1.72*	19.07**
Error 2	510	53.70	1.66	1.40	11.21
Coefficient of variation (%)		11.88	14.73	6.69	4.20

ns, *, **: non-significant and significant at five and one percent probability levels, respectively.

**Figure 1:**
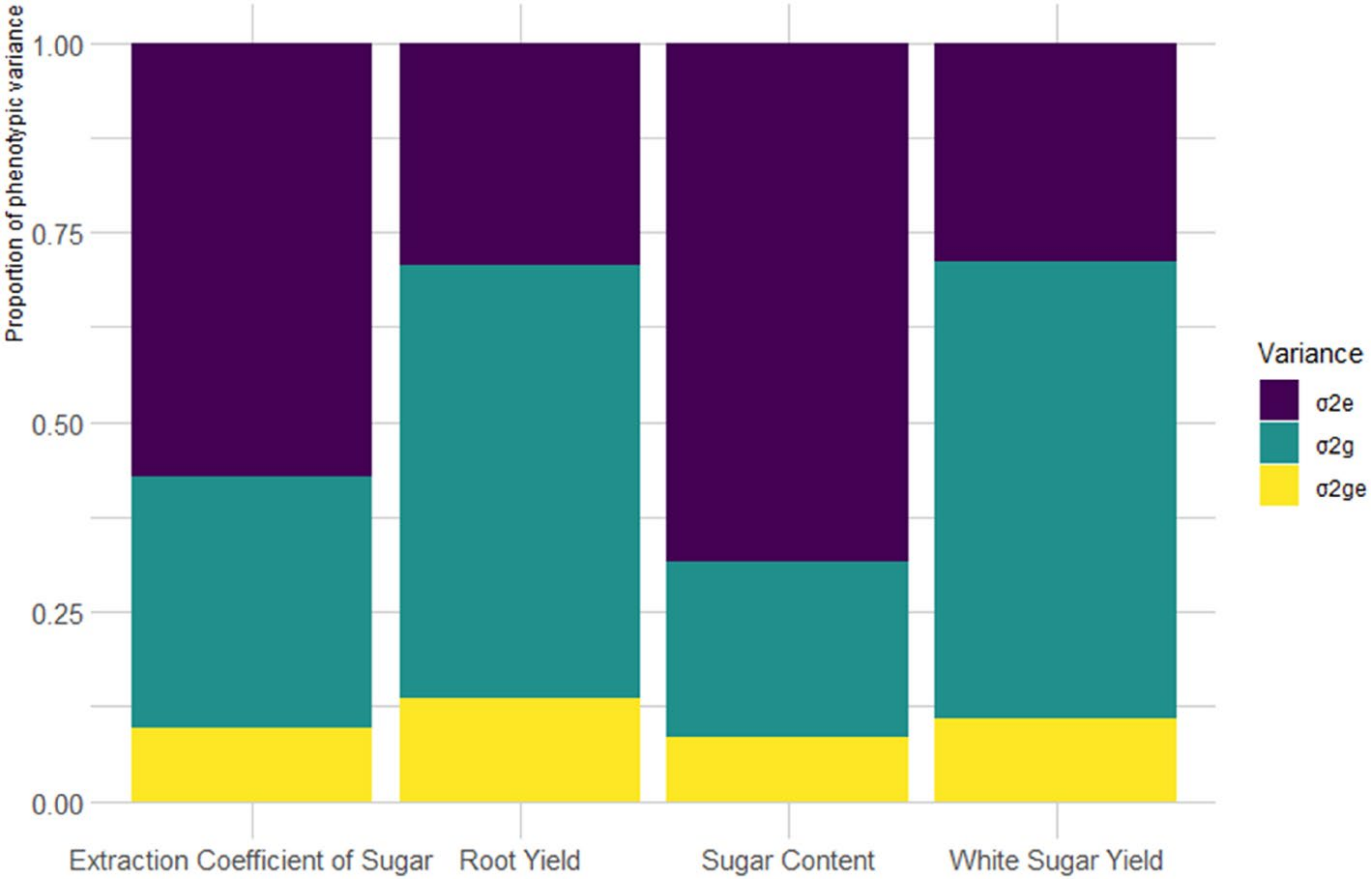
Proportion of the phenotypic variance for studied traits in sugar beet genotypes.

Phenotypic variance components including genetic variance, environmental variance and GEI variance was calculated for all traits (Fig. 1). Based on the results, significant genetic and environmental variance was observed for different traits. The highest phenotypic variance of RY and WSY was assigned to genetic variance and the highest phenotypic variance of SC and ECS was assigned to environmental variance. For RY and WSY, where the ratio of genetic diversity to environment is higher, the selection efficiency will increase and identification followed by selection of favorable genotypes from unfavorable ones will be conducted more accurately^26^. GEI variance had the lowest contribution to the phenotypic variance for all traits. In general, results indicated the high effect of genes in creating diversity among genotypes in terms of RY and WSY. Meanwhile, genotypes were affected by environmental conditions in terms of SC and ECS. The low variance of the GEI indicated the low effect of it on the phenotypic expression of traits in different genotypes, which caused low yield fluctuations from one environment to another. Contrary to the results of this study, Taleghani et al.^25^ reported high effect of environment (71.50%) on WSY followed by genotype (9.80%), and GEI (7.20%) for autumn-sown sugar beet. In Basafa and Taherian^27^ study, the justified variance by GEI was equal to 7.84%. Also, Mostafavi and Saremirad^28^ reported the justified variance for the environment, genotype, and GEI as 17.69%, 32.79%, and 17.90% of the total sum of squares, respectively. In their study, genotype caused the greatest variation in yield, which indicated high genotype diversity.

Considering the significant effect of GEI for all studied traits, multiplicative effects analysis was performed to identify stable genotypes based on AMMI's model. AMMI model with two significant IPCs is the best predicted model^29,30^. Based on the results (Table 5), the number of significant components varied from one to four for all studied traits. For RY, the first four components were significant at the 1% probability level and explained 91% of the variations related to GEI. The number of influencing components for WSY was equal to three, all of which were significant at the 1% probability level and explained 86.30% of the GEI effect. The variations related to the SC were justified by a significant component at the 1% probability level, which was equal to 56.40%. The ECS had two significant components that explained 60.66% of the GEI effect. The residual sum of squares from AMMI with the lowest mean of squares for all studied traits was non-significant, which indicates the considerable accuracy of this model^31^. In a study by Omrani et al.^32^ the first four components explained 83% of the GEI. Fathi et al.^33^ reported the contribution of the first and second PCs of the GEI as 49.49% and 22.50%, respectively, and both accounted for 71.60% of the variations in the GEI. In another study using AMMI model, the first IPC was significant and explained about 63% of GEI variation^28^. Rajabi et al.^23^ also reported the significant effect of first six IPCs explaining 98.80% of the total GEI variations.

**Table 5:** Multiplicative effects analysis of variance of AMMI model for studied traits in sugar beet genotypes.

Source of variation	df	Sum of squares	Mean of squares	Relative variance (%)	Cumulative variance (%)
Root yield (RY)					
IPCA1	25	21,908.23	876.32**	57.30	57.30
IPCA2	23	5792.93	251.86**	15.20	72.50
IPCA3	21	4551.09	216.71**	11.90	84.40
IPCA4	19	2523.79	132.83**	6.60	91.00
Noise	65	3425.29	52.69^ns^	9.00	100
White sugar yield (WSY)					
IPCA1	25	515.10	20.60**	53.40	53.40
IPCA2	23	186.46	8.10**	19.30	72.70
IPCA3	21	130.98	6.23**	13.60	86.30
Noise	84	132.59	1.57^ns^	13.70	100
Sugar content (SC)					
IPCA1	25	148.49	5.93**	56.40	56.40
Noise	128	114.59	0.89^ns^	43.60	100
Extraction coefficient of sugar (ECS)					
IPCA1	25	1379.25	55.17**	47.30	47.30
IPCA2	23	563.48	24.49**	19.30	66.60
Noise	105	974.23	9.27^ns^	33.40	100

ns, *, **: non-significant and significant at five and one percent probability levels, respectively.

**Figure 2:**
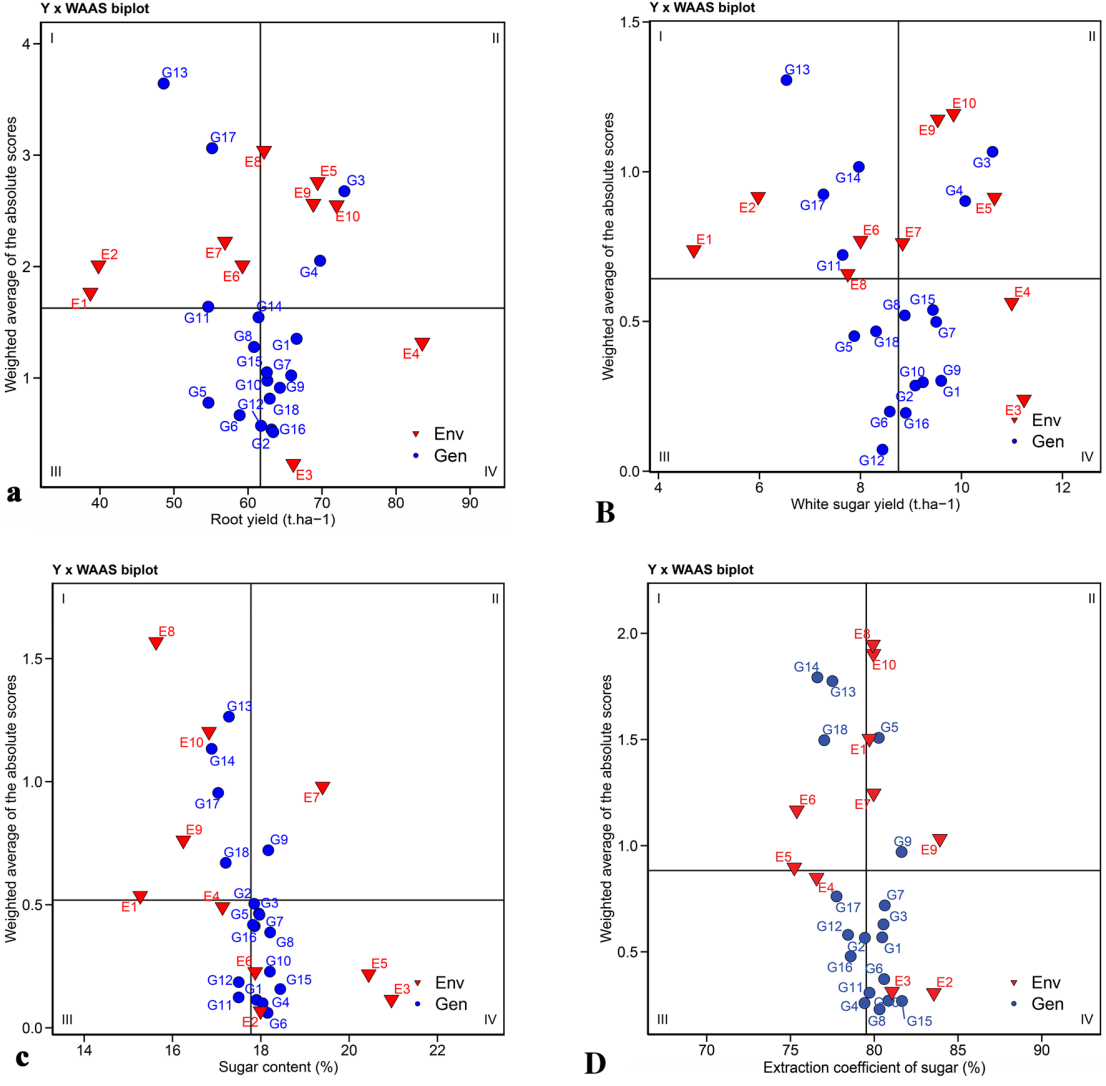
Mean performance vs. WAAS biplot from AMMI model for (**a**) root yield (RY), (**B**) white sugar yield (WSY), (**c**) sugar content (SC) and (**D**) extraction coefficient of sugar (ECS).

The biplot of mean performance versus WAAS which is called the WAAS biplot (Fig. 2), unlike AMMI model which considers only the first IPC, shows stability based on all scores of the IPCs. Therefore, WAAS considers the total variance of GEI in identifying stable genotypes. In this biplot, the vertical line in the middle of the biplot shows the total mean RY (Fig. 2a), WSY (Fig. 2B), SC (Fig. 2c) and ECS (Fig. 2D) of 10 experimental environments. Genotypes and environments on the right side of this line have a yield value higher than the total mean, and on the other hand, genotypes and environments on the left side of this line have a yield value lower than the total mean. The horizontal axis in the middle of the biplot shows the mean of the WAAS. From the intersection of this axis with the vertical axis, the biplot is divided into four quadrants. Genotypes in different quadrants of the biplot can be classified based on their suitability for different environments. The genotypes located in the first quadrant of the biplot include G13, G17 and G11 related to RY, G13, G14, G17 and G11 related to WSY, G13, G14, G17 and G18 related to SC and G14, G13 and G18 related to the ECS had a high WAAS value and yield value lower than the total mean, which indicates their highly fluctuating and unstable nature in terms of the relevant traits in different environments and a performance value lower than the mean. In general, these genotypes are not recommended for cultivation. Genotypes G3 and G4 located in the second quadrant of the RY and WSY biplots, G9 located in the second quadrant of the SC biplot, and G5 and G9 located in the second quadrant of the ECS biplot have high WAAS and performance value above the total mean. If the environmental conditions are favorable, the yield value of these genotypes will be high and can be recommended for cultivation in areas with ideal conditions for the growth and development of sugar beet. Genotypes G6, G5, G8 and G14 in the third quarter of the RY biplot, G12, G6, G5 and G18 in the third quarter of the WSY biplot, G11 and G12 in the third quarter of the SC biplot and G4, G16, G2, G12 and G17 in the third quarter of the ECS biplot had lower WAAS, which indicates their stability or lack of influence from environmental conditions. At the same time, these genotypes showed low performance value. Genotypes G2, G16, G18, G9, G10, G7, G15 and G1 located in the fourth quadrant of the biplot related to RY, G16, G2, G10, G1, G9, G7, G8 and G15 located in the fourth quadrant of the biplot related to WSY, G6,G 4, G1, G15, G10, G8, G16, G7, G5 and G3 located in the fourth quadrant of the biplot related to the SC and G8, G10, G15, G11, G6, G1, G3 and G7 located in the fourth quarter of the biplot related to the ECS had low WAAS and higher performance value than the total mean. Genotypes placed in this quadrant of the biplot are known as stable genotypes with optimal performance due to their low influence on environmental conditions as well as proper performance. In general, the WAAS biplot can be described in a way that genotypes with WAAS values of zero or close to zero are considered the most stable genotypes; Therefore, G2, G16, G12 and G6 in terms of RY, G12, G16 and G6 in terms of WSY, G6, G4, G1 and G11 in terms of SC, and G8, G10, G15, G4 and G11 in terms of ECS have low GEI and high stability, but the ideal genotypes are those that have a WAAS value of zero or close to zero and a yield value higher than the total mean. Genotypes G2 and G16 in terms of RY, G16 and G2 in terms of WSY, G6, G4 and G1 in terms of SC and G8, G10 and G15 in terms of ECS, in addition to stability, had a yield value higher than the total mean, so they were selected as stable genotypes with optimal performance value. Despite the different stability analysis methods, the AMMI provides useful information to achieve accurate results^28,34^.

### Best linear unbiased prediction (BLUP)

Based on the likelihood ratio test, the effect of genotype for all studied traits, including RY, WSY, SC, and ECS was significant at the 1% probability level (Table 6). However, GEI for RY, WSY, and ECS was found to be significant at 1% probability level and for SC at 5% probability level. The significance of GEI indicates the different response of genotypes and the superiority and weakness of them in different environments. Therefore, in such a situation, using the BLUP method can bring better and more reliable results^12^. Estimating traits heritability plays an important role in the breeding programs to identify and recommend genotypes^12,35^. Results showed high heritability for all traits. Among studied traits, WSY and RY had the highest heritability (Table 6). The correlation coefficient of GEI was low in all traits, especially the SC. Selection accuracy for WSY (0.92) and RY (0.90) was high, however for ECS (0.89) and SC (0.88) was in average (Table 6). This parameter shows the correlation between observed and predicted values^12^. The average and high selection accuracy values of the traits indicated the reliability of the model in selecting superior genotypes. The genotypic correlation between the environments was low in terms of all traits, while the SC had the lowest genotypic correlation between environments (Table 6). If low correlation exists, accurate information and details are needed to select superior genotypes^36^.

**Table 6:** Estimation of variance components from linear mixed model for studied traits in sugar beet genotypes.

	Root yield (RY)	White sugar yield (WSY)	Sugar content (SC)	Extraction coefficient of sugar (ECS)
Genotype	27.40**	0.90**	0.15**	1.90**
Genotype: environment	49.00**	1.16**	0.07*	1.96**
Residuals	53.70	1.66	1.42	11.20
Heritability	0.81	0.85	0.78	0.79
R^2^_GEI_	0.37	0.31	0.04	0.13
Accuracy	0.90	0.92	0.88	0.89
r_ge_	0.47	0.41	0.05	0.14

**Figure 3:**
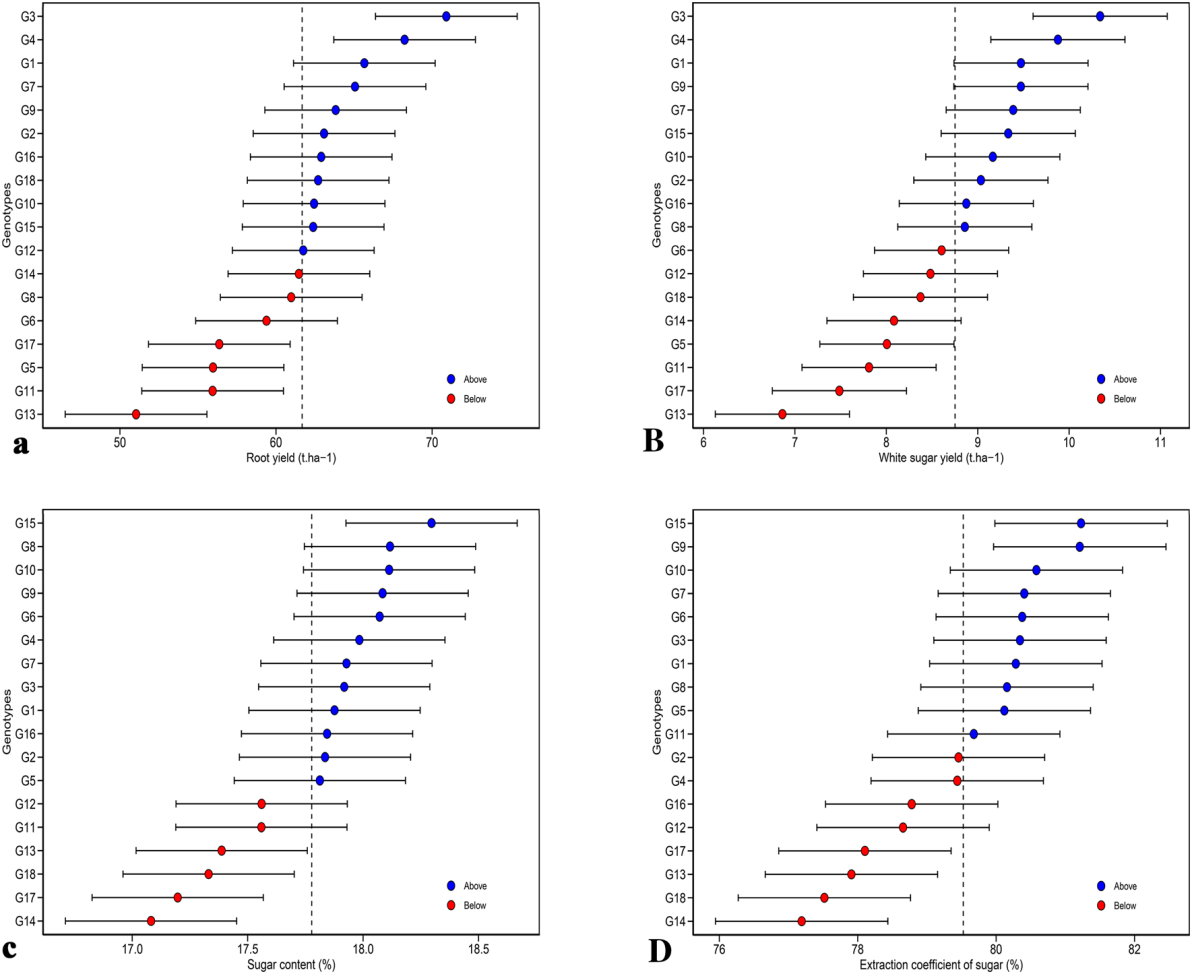
Best linear unbiased peridection mean values of sugar beet genotypes for (**a**) root yield (RY), (**B**) white sugar yield (WSY), (**c**) sugar content (SC) and (**D**) extraction coefficient of sugar (ECS).

The predicted mean values of the genotypes for each of the studied traits are presented in Fig. 3. Eleven genotypes had a higher than predicted mean value for RY, among which G3 and G4 had the highest predicted mean value of RY (Fig. 3a). Out of the total of 10 genotypes that had the predicted mean value of WSY above the total mean, G3, G4, G1 and G9 had the highest predicted mean (Fig. 3B). From a total of 18 studied genotypes, 12 genotypes had higher than predicted mean SC, but only G1 had the highest predicted SC values (Fig. 3c). Among the 10 genotypes that had a predicted mean value of ECS above the total mean, G15 and G9 had the highest predicted mean values (Fig. 3D). Genotypes G3 and G4 had the mean values of the BLUP model in terms of RY and WSY, which made them suitable genotypes in terms of the above-mentioned traits, but in terms of SC and ECS, G15 had the higher mean values of the BLUP. The BLUP method estimates the average performance of genotypes in mixed models with high efficiency. Therefore, it can fill the gap of AMMI regarding the analysis of LMM structure. The BLUP has been used in the evaluation of various crops such as rice^37^, corn^38^, cotton^39^ and sugarcane^40^ and useful results have been presented. One of the most important advantages of the BLUP method is the accurate estimation of the mean performance of genotypes, especially in LMMs^12,41,42^. In addition, when there is a LMM effect in the results, the BLUP allows optimal prediction of random effects^17,43^.

**Figure 4:**
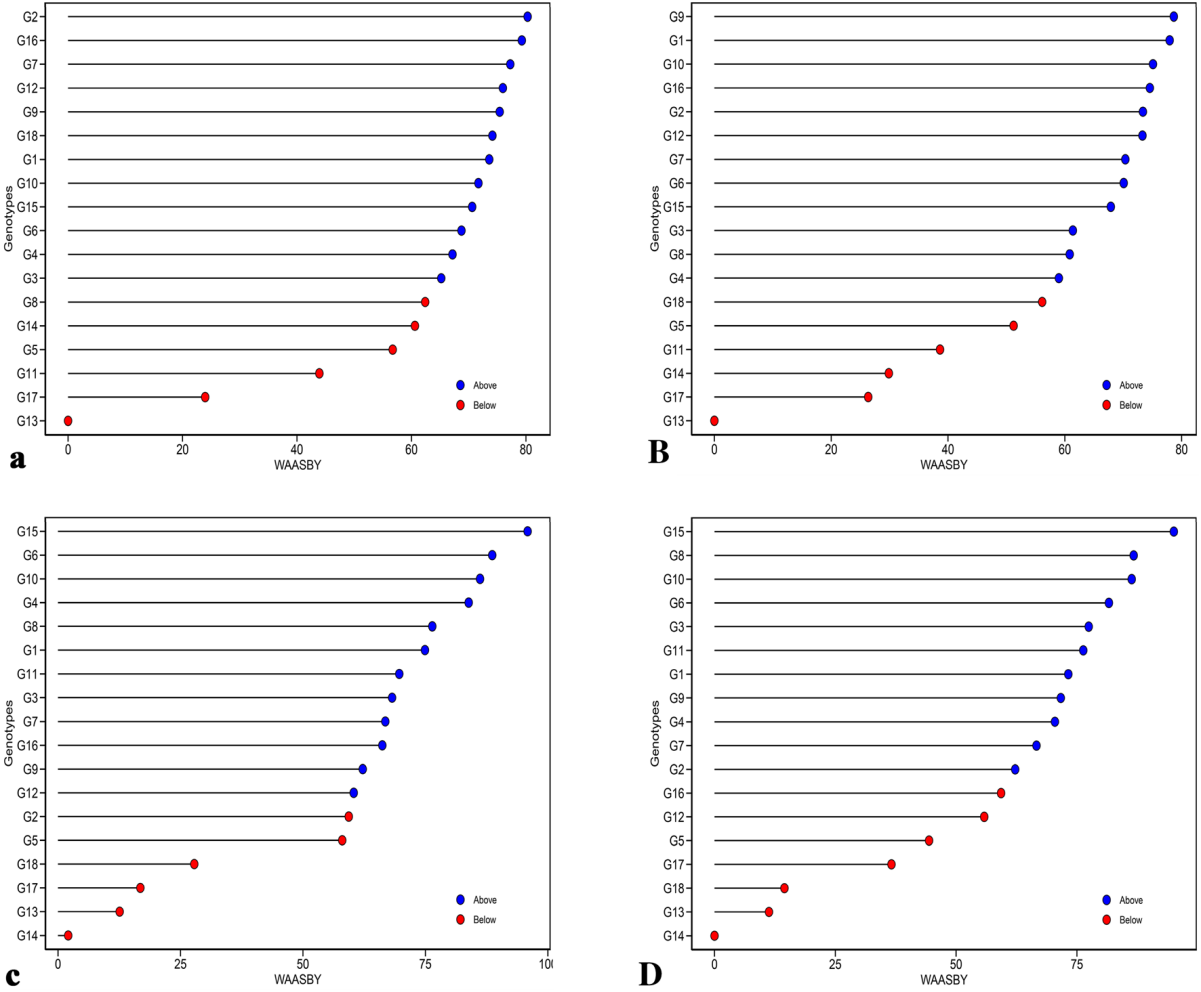
Estimated values of WAASBY index for (**a**) root yield (RY), (**B**) white sugar yield (WSY), (**c**) sugar content (SC) and (**D**) extraction coefficient of sugar (ECS) in sugar beet genotypes considering the weights of 50 (yield) and 50 for (stability).

In Fig. 4, it is possible to rank and select genotypes at the same time based on yield value and stability. The WASSBY is actually a combination of the WAASB and the yield value of the trait. Blue circles indicate higher than mean WAASBY and red circles indicate lower than mean WAASBY index. Regarding RY, G12 had WAASBY index higher than the mean and G2 and G16 had relatively higher WAASBY index values compared with other genotypes (Fig. 4a). Based on this, G2 and G16 were recognized as stable genotypes with high RY. Results of the WAASBY for the WSY showed that among 12 genotypes with an index higher than the mean, G9 and G1 were the best in terms of higher values compared with other genotypes (Fig. 4B). Twelve genotypes for SC (Fig. 4c) and 11 genotypes for ECS (Fig. 4D) had high WAASBY. Genotype G15 was selected as the best genotype in terms of both above-mentioned traits. Stability parameters are important for breeders to ensure the stability of quality properties throughout different environments and influences. Olivoto et al. presented the WAASBY index, in which both stability and performance characteristics are considered simultaneously. Based on the results, stable genotypes could be identified successfully by this index.

### GGE biplot graphical analysis

In order to identify the variation between genotypes, environments and to study the GEI, GGE biplot graphical analysis method was used. Results of the GGE biplot showed that the first and second PCs accounted for 68.71% and 10.45% (a total of 79.16%) of the variations in RY, respectively. For WSY, the first two PCs accounted for 70.08% and 12.05%, respectively and for SC, for the contribution was 61.62% and 10.09%, respectively. However, for ECS, it was 59.47% and 12.79%, respectively. Since the first two PCs accounted for a considerable rate of variance for all studied traits, it can confirm the relatively high validity of the biplot obtained from this study in explaining the variations of genotype and GEI. If the sum of the first and second PCs cannot explain most of the data variation, it indicates the complex nature of the GEI^44^, but this does not mean that the biplot is invalid^15^. According to statements, when biplot justify at least 60% of the data variance, it can be used to determine MEs^45^. Hassani et al.^46^ showed that 62.9% of the GEI variations are explained by the first two PCs. In a study, Saremirad et al.^47^ estimated the sum of the first and second PCs to be nearly 74% with first and second PCs accounted for about 46% and 27% of the total variation, respectively.

**Figure 5:**
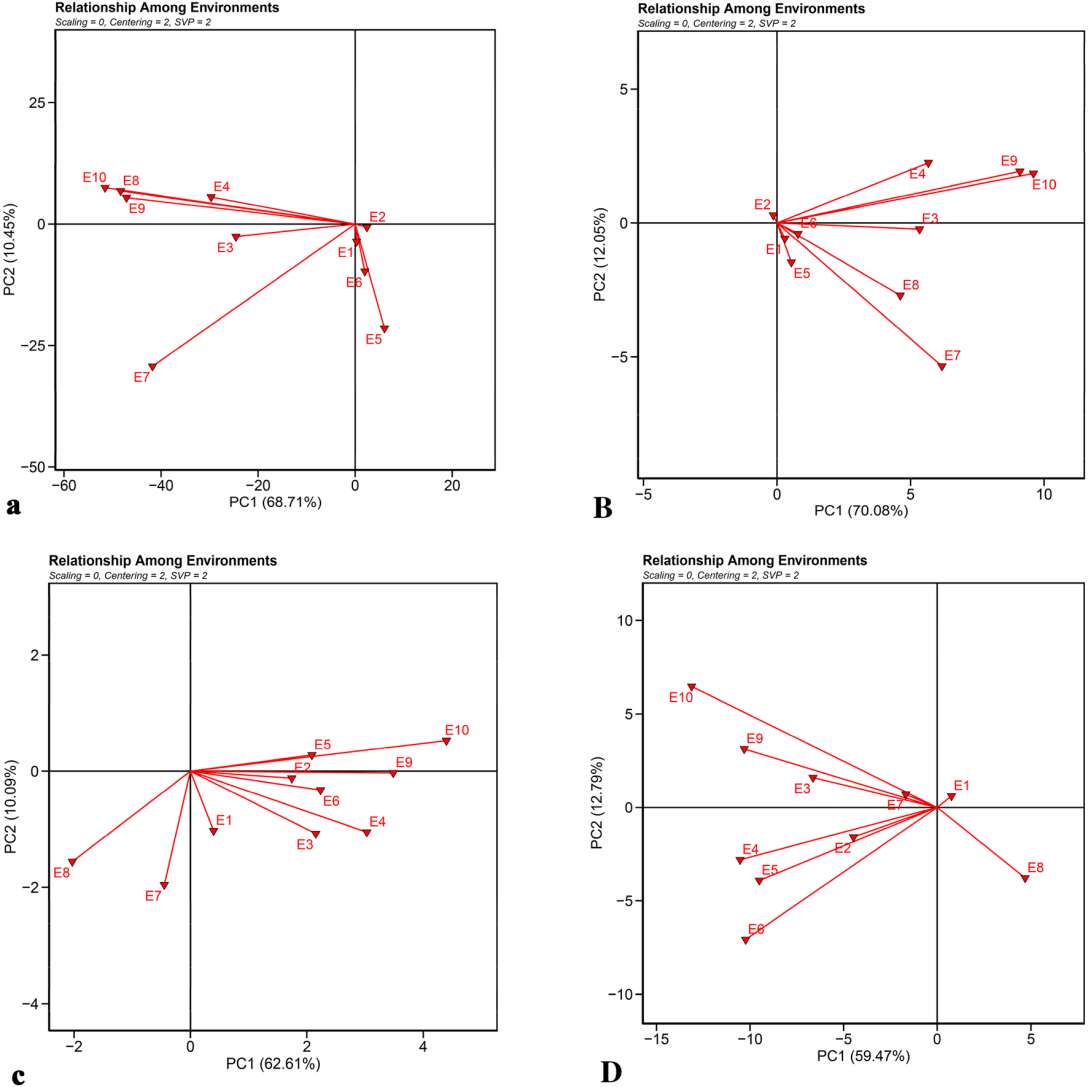
Biplot examine the relationship between the environments based on (**a**) root yield (RY), (**B**) white sugar yield (WSY), (**c**) sugar content (SC) and (**D**) extraction coefficient of sugar (ECS).

Figure 5 shows the correlation and relationships between environments. Evaluating the correlation between the studied environments not only show their relationships but also can contribute in planning future trial’s location in terms of time and money. In Fig. 5, if the angle between the vectors of the environments is smaller, the more correlation is among them. In fact, a high correlation between environments means a high correlation between the rank of genotypes in those environments. As shown in the RY graph (Fig. 5a), there is a high positive correlation between Karaj (2020) and Shiraz (2020 and 2021), among Mashhad (2020 and 2021), Hamedan (2020 and 2021) and Miandoab (2021), which indicates the relatively similar response of genotypes in these regions. Therefore, there is a small difference in genotype ranking for RY. The environmental conditions at the Karaj showed no correlation during 2 years of the study which shows that the genotypes response in this station was independent in both years. The biplot of WSY (Fig. 5B) showed that there was a positive correlation between Mashhad and Hamadan and also among Shiraz, Miandoab and Karaj in 2020. Karaj (2021) had a negative correlation with all environments. In terms of SC (Fig. 5c), there was a high correlation among Hamedan, Mashhad, Shiraz and Karaj in 2021 and between Miandoab and Karaj in 2020. In terms of ECS (Fig. 5D) there was a high correlation among Hamadan, Mashhad in 2020 and Miandoab (2020), and also among Shiraz, Mashhad in 2021 and Karaj (2021). Each of the environments of Karaj in 2020 and Miandoab in 2021 showed zero to negative correlation with the rest of the environments in terms of ECS. The length of the environment vector is an approximation of the standard deviation within each environment and is also an indicator for the differentiation of environments, so that environments with a longer vector length have a greater standard deviation and therefore have a greater ability to differentiate^48^; Therefore, one of the important features of any environment is its ability to differentiate, so that environments lacking the ability to differentiate cannot provide useful information about the studied genotypes^48^. Evaluation of the environment vectors indicated that Hamedan, Miandoab and Mashhad have long vector lengths which indicate the high discriminability of these environments, and the Karaj environment had less discriminability due to its shorter vector length. Finally, the biplot study of the environment correlation showed that most of the studied environments had a high discrimination ability and can create a suitable distinction between genotypes.

**Figure 6:**
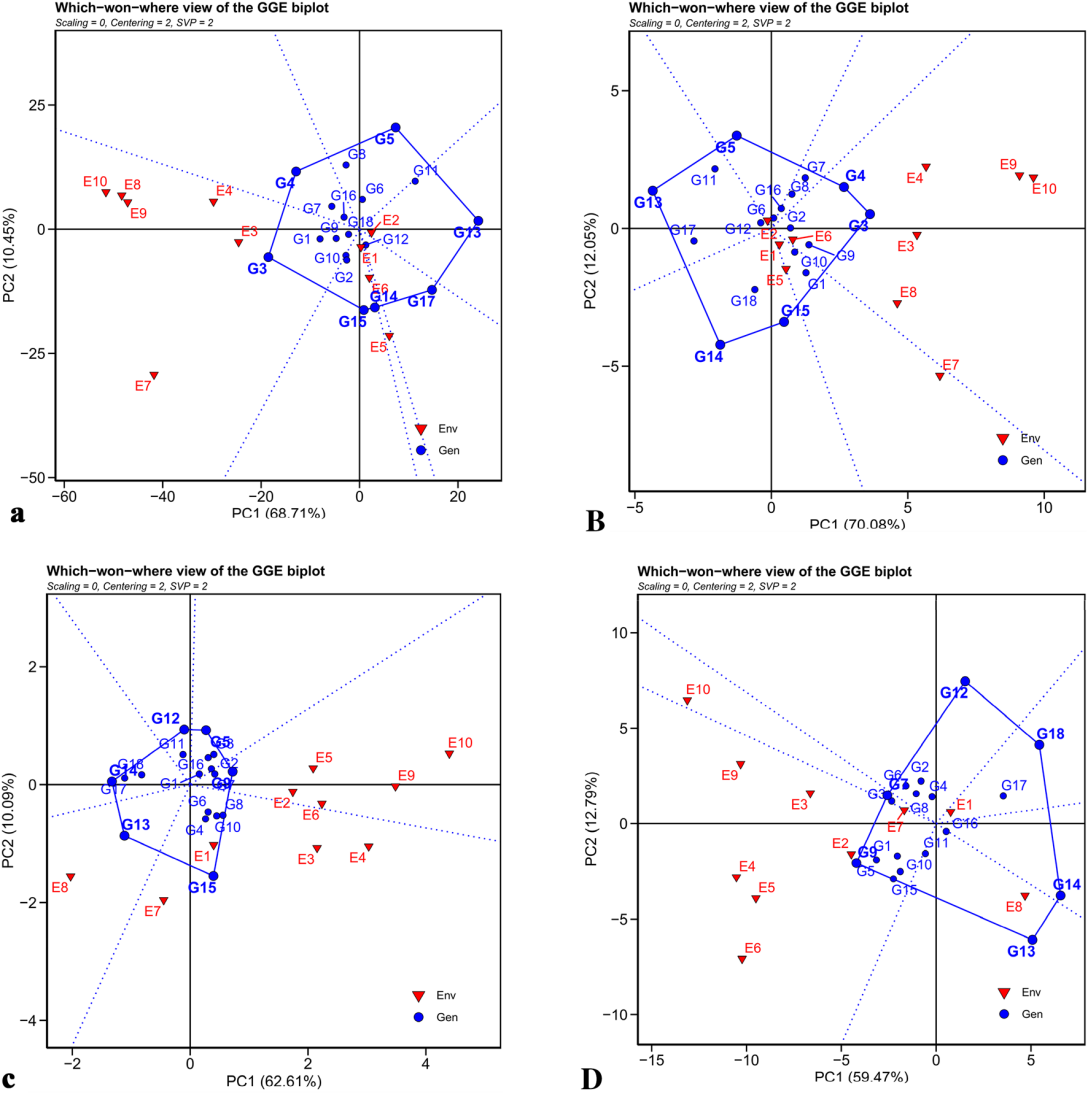
Polygons of GGE biplot method for determine the appropriate genotypes in every environment based on (**a**) root yield (RY), (**B**) white sugar yield (WSY), (**c**) sugar content (SC) and (**D**) extraction coefficient of sugar (ECS).

Figure 6 is related to the polygonal biplot of studied traits, which was drawn to identify MEs and superior genotypes in different environments. In this biplot, a polygon is drawn from the connection of the genotypes that have the maximum distance from the coordinate origin. Genotypes G5, G13, G17, G14, G15, G3 and G4 in Fig. 6a, G4, G3, G15, G14, G13 and G5 in Fig. 6B, G5, G9, G15, G13, G14, and G12 in Fig. 6c, and G12, G18, G14, G13, G9, and G7 in Fig. 6D were located at the farthest distance and formed a polygon. Then, from the origin of the coordinates, perpendicular lines were drawn on the sides of this polygon and the MEs were determined^15^. In the sections where the environments were placed and there are genotypes at the top of them, it means that these genotypes have optimum performance in those environments. Genotype G13 in Karaj (2021), G14 in Shiraz, G15 in Karaj (2020), and G3 in Mashhad, Miandoab and Hamedan were the best genotypes in terms of RY. In terms of WSY, G3 and G4 in Hamadan, Mashhad, Miandoab (2021), and Shiraz (2021), G15 in Miandoab (2020), Shiraz (2020) and Karaj (2020) and G5 in Karaj (2021) were the best and stable genotypes. Genotype G9 in the Hamedan, Shiraz and Karaj (2021), G15 in Mashhad, Karaj (2020) and Miandoab (2020), and G13 in Miandoab (2021) had the highest SC. The highest ECS was observed in G18 in Karaj (2020), G13 in Miandoab (2021), Mashhad, Shiraz, Miandoab (2020), Hamedan (2020), G9 in Karaj (2021), and G7 in Hamedan (2021). Genotypes placed in sections where there is no environment are not desirable for cultivation in any of the studied environments and are among the weak genotypes in most of the environments. Based on the polygonal biplot, the experimental environments were studied in different MEs in terms of different traits. So that the experimental environments were grouped in terms of RY and ECS in four MEs and in terms of WSY and SC in three MEs. Using a polygonal biplot, Hassani et al.^49^, classified different sugar beet environments into two groups and introduced the genotypes with the highest adaptability. Saremirad and Taleghani^24^ evaluated sugar beet hybrids in seven regions in terms of stability. Their results showed that the GEI overshadows the quantitative and qualitative characteristics of sugar yield in hybrids, so this issue should be considered when breeding new hybrids. Estimation of GEI provides the possibility to make a decision regarding the breeding for general or specific adaptation that depends on the stability of performance in a limited or wide range of environmental conditions and an effective step should be taken towards the development of cultivars with stability and adaptability with the target environments.

### Multi-trait stability index (MTSI)

**Figure 7:**
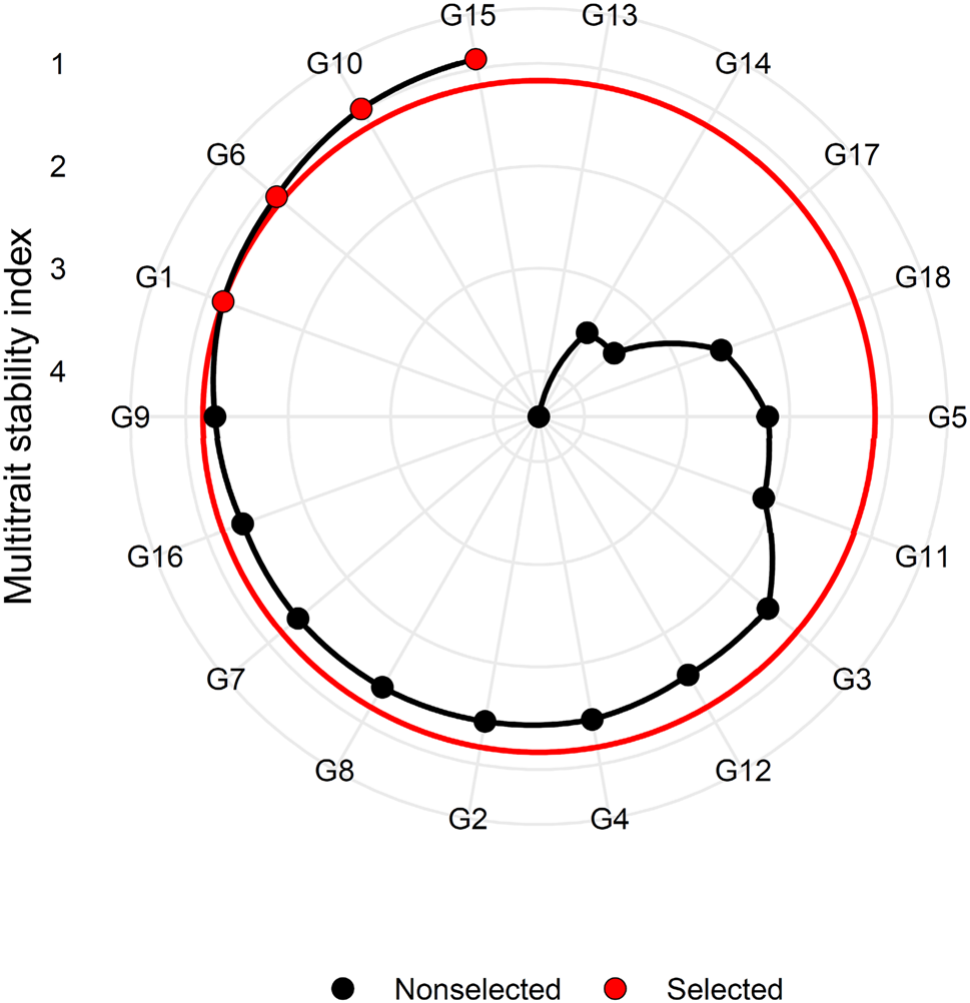
Ranking of sugar beet genotypes in ascending order based on MTSI index.

MTSI was computed based on quantitative and qualitative traits. In Fig. 7, the experimental genotypes are ranked from the highest to the lowest value of the MTSI so that the genotype with the highest value of MTSI is in the center and the genotype with the lowest value of MTSI is located in the outermost circle. The genotypes determined in red color dots were selected based on their MTSI values at 20% selection intensity. G15 was in the first rank followed by G10, G6 and G1 as the most ideal stable genotypes. Average value of all traits in selected genotypes has increased which was aimed at the intended goals. In general, the selected genotypes caused favorable selection differential in all traits (Table 7). Sharifi et al.^50^ and Koundinya et al.^36^ reported that MTSI would be very useful to the plant breeders for the selection of superior genotypes for multiple traits based on multi-environment data. Zuffo et al.^51^ used MTIS to identify stable soybean genotypes under drought and salinity stress conditions. Based on the MTSI results, Rajabi, et al.^23^ introduced five sugar beet genotypes as stable genotypes under field condition infected with rhizomania disease. These results were consistent with the findings obtained from this study regarding the efficiency of MTSI in identifying superior genotypes.

**Table 7:** Prediction of selection differential for studied traits based on MTSI index.

Row	Traits	Factor 1	Goal	Xo	Xs	SD	SD percent	h^2^
1	Root yield (RY)	− 0.82	Increase	61.67	62.65	0.98	1.59	0.81
2	White sugar yield (WSY)	− 0.94	Increase	8.75	9.21	0.46	5.27	0.85
3	Sugar content (SC)	− 0.92	Increase	17.78	18.18	0.40	2.24	0.78
4	Extraction coefficient of sugar (ECS)	− 0.87	Increase	79.52	80.89	1.37	1.72	0.80

*Xo* original value, *Xs* selected value, *SD* selection differential, *SD perc* selection differential in percentage, *h*^*2*^ broad sense heritability.

## Conclusion

GEI was significant for studied quantitative and qualitative traits in terms of the used stability analysis methods. It can be concluded that the environment has played a significant role in influencing the phenotypic expression of quantitative and qualitative traits of sugar beet especially for SC an ECS. Although, the highest phenotypic variance of RY and WSY was assigned to genetic variance, these traits also showed the highest heritability and selection accuracy. According to the results, G2 and G16 in terms of RY, G16 and G2 in terms of WSY, G6, G4, and G1 in terms of SC and G8, G10 and G15 in terms of ECS were known as stable genotypes with favorable performance. In general, G15 was the most ideal genotype in terms of all traits followed by G10, G6 and G1. Based on the environmental conditions of the experimental areas and their effect on the growth and development of sugar beet, these areas are among the important and major areas for sugar beet cultivation. Experimental areas were grouped in terms of RY and ECS into four MEs and in terms of WSY and SC into three MEs. In general, by applying various stability analysis methods, it not only contributes to evaluate the different aspects of GEI nature but also stable genotypes are selected more accurately. In this regard, the MTSI can be useful due to its power in the simultaneous selection of stable genotypes based on multi traits.

## Data Availability

The data that support the findings of this study are available from the corresponding author upon reasonable request.
